# Detection of the location of pneumothorax in chest X-rays using small artificial neural networks and a simple training process

**DOI:** 10.1038/s41598-021-92523-2

**Published:** 2021-06-22

**Authors:** Yongil Cho, Jong Soo Kim, Tae Ho Lim, Inhye Lee, Jongbong Choi

**Affiliations:** 1 Department of Emergency Medicine, College of Medicine, Hanyang University, 222 Wangsimni-ro, Seongdong-gu, Seoul, 04763 Republic of Korea; 2 Institute for Software Convergence, Hanyang University, 222 Wangsimni-ro, Seongdong-gu, Seoul, 04763 Republic of Korea; 3 Department of Emergency Medicine, Yonsei University College of Medicine, Seoul, Republic of Korea; 4 Department of Biomedical Engineering, Hanyang University, Seoul, Republic of Korea

**Keywords:** Health care, Medical research, Mathematics and computing

## Abstract

The purpose of this study was to evaluate the diagnostic performance achieved by using fully-connected small artificial neural networks (ANNs) and a simple training process, the Kim-Monte Carlo algorithm, to detect the location of pneumothorax in chest X-rays. A total of 1,000 chest X-ray images with pneumothorax were taken randomly from NIH (the National Institutes of Health) public image database and used as the training and test sets. Each X-ray image with pneumothorax was divided into 49 boxes for pneumothorax localization. For each of the boxes in the chest X-ray images contained in the test set, the area under the receiver operating characteristic (ROC) curve (AUC) was 0.882, and the sensitivity and specificity were 80.6% and 83.0%, respectively. In addition, a common currently used deep-learning method for image recognition, the convolution neural network (CNN), was also applied to the same dataset for comparison purposes. The performance of the fully-connected small ANN was better than that of the CNN. Regarding the diagnostic performances of the CNN with different activation functions, the CNN with a sigmoid activation function for fully-connected hidden nodes was better than the CNN with the rectified linear unit (RELU) activation function. This study showed that our approach can accurately detect the location of pneumothorax in chest X-rays, significantly reduce the time delay incurred when diagnosing urgent diseases such as pneumothorax, and increase the effectiveness of clinical practice and patient care.

## Introduction

Pneumothorax is a common thoracic disorder involving an abnormal collection of air in the space between the lungs and the chest wall^1^. A large volume of air in the pleural cavity can lead to tension pneumothorax, which compress the lungs and heart, causing shock and often death^2^. Therefore, it is important to check for the presence or absence of pneumothorax by taking a chest X-ray when a patient complains of chest pain and dyspnea. Although the diagnosis of pneumothorax based on chest X-rays is essential for patient safety, doing so is difficult for physicians and requires complex imaging techniques^3^. A number of studies have been conducted regarding the use of deep learning for pneumothorax classification with chest X-rays^4–8^. However, existing studies using deep- learning methods to detect the location of pneumothorax in chest X-rays are insufficient^9–11^, where these studies have used the convolution neural networks^12^ (CNNs).

Completely different from the back-propagation method^13–15^ which is the most commonly used method to date for training artificial neural networks (ANNs), a novel deep-learning algorithm, namely, the Kim-Monte Carlo algorithm, has been recently reported^16^. An author (J. S. Kim) developed this algorithm, which imitates the biological evolution of animals that adapt to a given environment according to the principle of the survival of the fittest^16^. The algorithm, a simple training process for ANNs, has been applied to predict the location of the glottis in laryngeal images^16^, to detect and classify intracranial haemorrhage on CT images^17^, and to detect pneumoperitoneum in abdominal radiograph images^18^. For data classification problems, there have also been studies using ANNs containing morphological neurons^19,20^.

The purpose of this study was to investigate the diagnostic performances of various fully-connected small ANN models for detecting the location of pneumothorax in chest X-rays using a simple training process (the Kim-Monte Carlo deep-learning algorithm). The dependence of diagnostic performance (in terms of detecting the location of pneumothorax) on the resolutions of the X-ray images input into the ANNs was also studied^16,18^. In addition, a common deep-learning method for image recognition, CNN, was also applied to the same dataset for comparison purposes.

## Methods

### Database

We conducted a study using NIH (the National Institutes of Health) Chest X-rays dataset (available at https://nihcc.app.box.com/v/ChestXray-NIHCC). The institutional review board (IRB) of Hanyang University Seoul Hospital (Seoul, Republic of Korea) approved this study and confirmed that all methods in this study were performed in accordance with the Good Clinical Practice guidelines with the need for informed consent waived (IRB No. HYUH 2021-03-024).

The NIH Chest X-rays dataset contains 112,120 frontal chest X-rays from 30,805 patients. These X-ray images have text-mined labels with 14 common thorax disease extracted from relevant radiological reports^21^. These labels were obtained through natural language processing and are inherently inaccurate^22^. For example, there are 5302 X-ray images labeled as pneumothorax, among which are a mixture of images with and without pneumothorax. Therefore, we randomly arranged the 5302 images using Microsoft Excel. Then, two board-certified emergency medicine specialists (Y. Cho and I. Lee) identified the images labeled with pneumothorax and sequentially selected up to 1000 grayscale chest X-ray images actually contained pneumothorax. The 1000 chest X-ray images consisted of 690 posterior-anterior images and 310 anterior- posterior images, without lateral images. Among the 1000 images, the findings accompanying pneumothorax were as follows: effusion 174 cases, infiltration 170 cases, emphysema 164 cases, atelectasis 132 cases, mass 69 cases, nodule 64 cases, pleural thickening 43 cases, consolidation 31 cases, fibrosis 11 cases, pneumonia eight cases, cardiomegaly seven cases, edema five cases, and hernia two cases. The X-ray images were used as the training data (80%) for two types of deep-learning methods, i.e., fully-connected small ANNs and CNNs, and the test data (20%) were used as a reference for comparing the detection results of deep-learning models. Therefore, one thousand chest X-ray images with pneumothorax were randomly divided into 800 cases for the training set and 200 cases for the test set.

### Preparing image data

**Figure 1:**
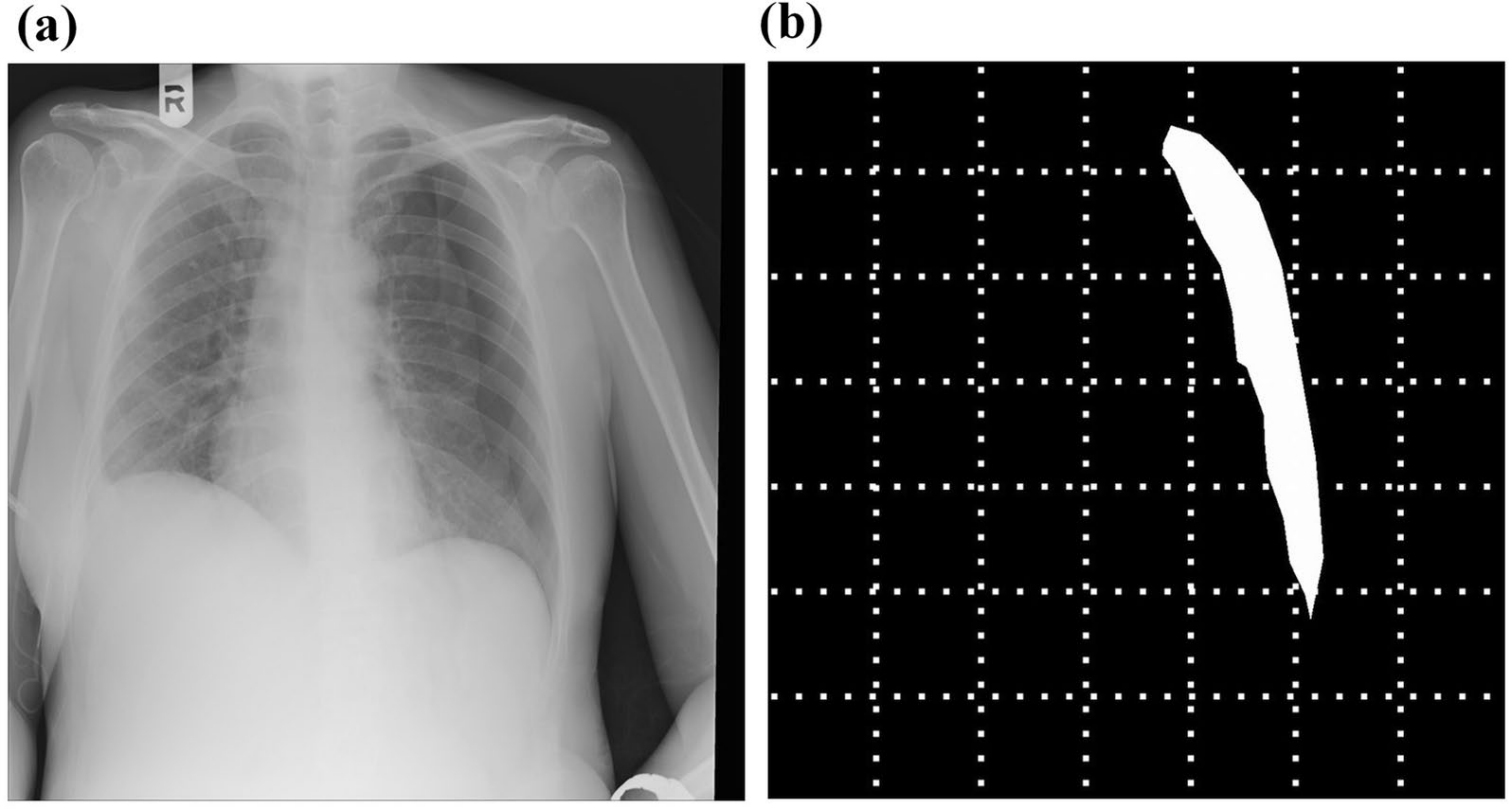
Example of chest X-ray image processing for deep-learning methods. (**a**) Original chest X-ray image. (**b**) Image obtained after marking the location of pneumothorax in white and the remaining areas in black.

Two board-certified medical experts (Y. Cho and I. Lee) with 12 and 8 years of experience as emergency medicine doctors, respectively, reviewed the chest X-ray images and marked the location of pneumothorax with a consensus. They work in the emergency department of university hospital. In Korea, chest X-rays are routinely taken for patients visiting the emergency room. Therefore, they check thousands of chest X-ray images of various aspects each year in clinical practice. ImageJ software (from National Institutes of Health, Maryland, USA) was used for marking the 1000 chest X-ray images with pneumothorax. The areas with pneumothorax were marked in white, and the rest of the images were marked in black (see Fig. 1). Each pneumothorax-marked image was then divided into seven horizontal sections and seven vertical sections. This segmentation process yielded 49 boxes per pneumothorax-marked image, as shown in Fig. 1b. The target values of the boxes containing one or more white pixel values were set to 1, and the target values of the other boxes were set to 0.

### Structures of the ANN and CNN models

To obtain a fully-connected small ANN structure with the optimal diagnostic performance, the input image resolution and the number of hidden layers in the ANN model started at 20 × 20 and 1, respectively, and were gradually increased^17,18^ to compare the resulting performances with those of other ANN models. Therefore, the pixel resolution of the original chest X-ray images (512 × 512) was reduced to 20 × 20, 30 × 30, 40 × 40, 60 × 60, and 80 × 80 pixels. The number of input nodes for an ANN should be the total number of pixels in the given image at a reduced resolution. Thus, each black-and-white pixel value was divided by the maximum value of 255 to achieve a value between zero and 1.0 for use as the input value of an input node^16^. The number of output nodes of ANN was set to 49 (see Fig. 1b) for detecting the pneumothorax location. The number of hidden layers of a fully-connected ANN was set to 1, 2, or 3. The number of nodes in the first hidden layer was set to 49, 98, or 196. Accordingly, 45 fully-connected small ANN models (= 5 resolutions × 3 × 3) were constructed.

**Figure 2:**
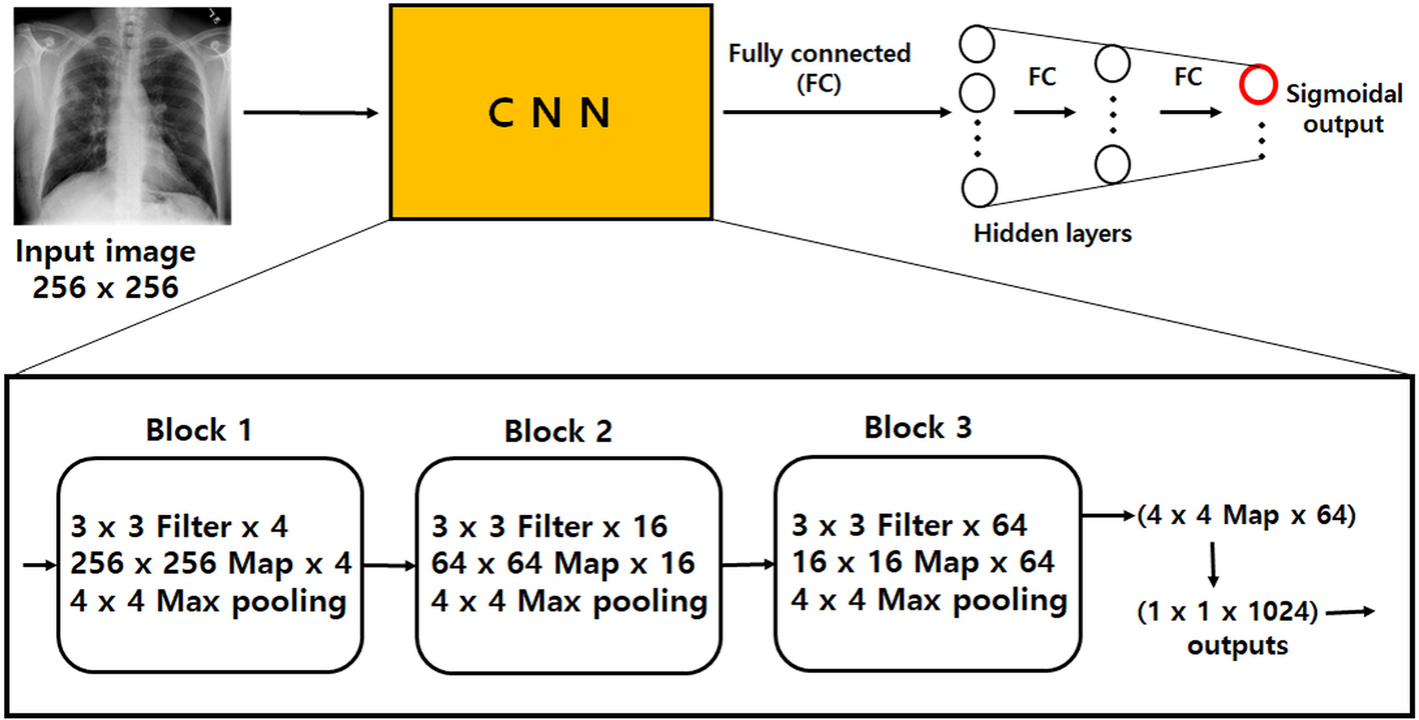
A detailed schematic representation of the actual architecture of CNN.

A CNN^12^ was also applied to the same dataset with an input pixel resolution of 256 × 256. Figure 2 shows a detailed schematic representation of the actual architecture of the CNN. As shown in Fig. 2, the CNN structure consisted of a convolution part and a fully-connected part. In the block 1 of the convolution part, an input image of 256 × 256 pixels was applied to four 3 × 3 filters with stride of 1 and zero-padding with 1; 4 separate activation maps of 256 × 256 were generated with the rectified linear unit (RELU) activation; and the activation maps of 256 × 256 were converted into 4 individual activation maps of 64 × 64 by 4 × 4 max-pooling with stride of 4. In the block 2, each of the 4 individual activation maps of 64 × 64 was applied to four 3 × 3 filters with stride of 1 and zero-padding with 1; 16 separate activation maps of 64 × 64 were generated with RELU activation; and the activation maps of 64 × 64 were converted into 16 individual activation maps of 16 × 16 by 4 × 4 max-pooling with stride of 4. In the block 3, each of the 16 individual activation maps of 16 × 16 was applied to four 3 × 3 filters with stride of 1 and zero-padding with 1; 64 separate activation maps of 16 × 16 were generated with RELU activation; and the activation maps of 16 × 16 were converted into 64 individual activation maps of 4 × 4 by 4 × 4 max-pooling with stride of 4. Then, the output of the convolution part, 4 × 4 × 64, was converted into an input for the fully-connected part with a size of 1 × 1 × 1024. Thus, the convolution part contained a total of 84 individual 3 × 3 convolution filters (= 4 + 16 + 64) with 3 × 3 × 84 weight factors and 84 bias values. The number of hidden layers in the fully-connected part was set to 1 or 2. The number of hidden nodes was set to 49 or 49–49. The number of output nodes in the fully-connected part was set to 49 (see Fig. 1b) for detecting the pneumothorax location.

### ANN and CNN training process

The Kim-Monte Carlo algorithm^16^, a simple ANN training process that differs from the back-propagation method^13–15^, was applied to train the various fully-connected small ANN models and the CNNs. The algorithm applied a random optimization process based on a Monte Carlo simulation during the training session to determine a very large number of unknown weight factors and bias values for each ANN or CNN with variables that minimized the average training error for a given training dataset. The initial weight factors and bias values were randomly chosen within ranges of -0.2 to + 0.2 and 0 to + 0.2, respectively^16^. The algorithm consisted of (a) randomly selecting the weight factors and bias values based on a given variable selection ratio and adjusting their values by small random amounts (within a range of -0.1 to + 0.1^16^), where the bias value was changed to positive if the variable value was changed to negative; (b) accepting or rejecting the adjustments depending on whether the new values decreased the average training error for all training data; and (c) repeating the above two steps^16^. A training session was terminated after 10 repetitions of the training cycle, during which the variable selection ratio of the training cycle steadily decreased from 15 to 1.5%^16^ of the total number of variables in the ANN or CNN. During the training cycle, the total sum of the variable selection ratios was set to 900%, and 30 attempts were made to adjust the values of the selected variables by small random amounts^17,18^. After the training session was completed, the test set was applied to the ANN or CNN to obtain the test results and identify the diagnostic performance of the deep-learning model.

For fully-connected hidden nodes, a sigmoid or RELU activation function was applied.

1 $$ \begin{gathered}   y_{j}  = x_{j} ,\quad {\text{if }} x_{j}  > 0 \hfill \\   y_{j}  = 0, \quad {\text{if }}x_{j}  \le 0 \hfill \\  \end{gathered} $$

The RELU activation function for fully-connected hidden nodes was implemented using Eq. (1), and the sigmoid activation function was implemented by the following formulas:

2 $$ y_{j}  = \frac{2}{{1 + e^{{ - x_{j} }} }} - 1 $$

3 $$ y_{j}  = \frac{1}{{1 + e^{{ - x_{j} }} }} $$

4 $$ x_{j}  = \mathop \sum \limits_{i} w_{{ij}} y_{i}  + b_{j} $$

where Eq. (4) (using one of Eqs. (1), (2), and (3)) denotes the summation over all nodes in the previous layer, i.e., each node *j* in a given layer receives an input ${y}_{i}$ from a node *i* in the previous layer; ${w}_{ij}$ indicates the weight factor between nodes *i* and *j*; and ${b}_{j}$ indicates the bias value of a node *j*. The sigmoid activation functions in Eqs. (2) and (3) were applied for fully-connected hidden nodes and output nodes, respectively.

Details about the hardware and software infrastructure used to implement the ANNs and CNNs are described below. The software for applying the novel deep-learning algorithm, a simple training process, to the ANNs and CNNs was developed and programmed fully in-house by J. S. Kim using Microsoft Visual C +  + . Regular PCs without graphics processing unit (GPU) computing were used as the hardware. The operating system used was Microsoft Windows 10 64-bit professional, and the CPU was Intel i5-7400. The main memory size was 16 GB. The time it took to train a small ANN with the training set depended on the size of the ANN structure, and it took 3 to 5 h for the training set with 800 chest X-ray images. It took approximately one second to predict the entire test set of 200 X-ray images. The time it took to train a CNN was approximately 50 h for the training set of 800 chest X-ray images of an input resolution of 256 × 256 pixels. It took a few seconds to predict the entire test set.

### Statistical analysis

We performed a receiver operating characteristic (ROC) curve analysis to determine the diagnostic performances of various ANNs and CNNs with respect to pneumothorax localization. The area under the ROC curve (AUC) value for each network was obtained, and the cut-off was determined using the Youden index. Statistical analyses were conducted using R software [version 4.0.1] (R: A Language and Environment for Statistical Computing, R Core Team, R Foundation for Statistical Computing, Vienna, Austria, 2021, http://www.R-project.org).

## Results

Table 1 shows the pneumothorax detection results for each of the boxes in the chest X-ray images of the test set achieved by the small ANN models including 1, 2, or 3 hidden layers. The results were selected by comparing the test results among the 45 fully-connected ANN models according to the input resolutions of the chest X-ray images, i.e., 20 × 20, 30 × 30, 40 × 40, 60 × 60, and 80 × 80 pixels. In Table 1, PPV indicates the predictive values of the positive test, and NPV indicates the predictive values of the negative tests. By using the fully-connected ANN structure including 900 input nodes (for an input pixel resolution of 30 × 30), 49 intermediate nodes in the first hidden layer, 49 intermediate nodes in the second hidden layer, 49 intermediate nodes in the third hidden layer, and 49 output nodes, we obtained the best diagnostic performance among those of the 45 ANN models with sigmoid activation functions [refer to Eqs. (2) and (3)] for all nodes; the AUC was 0.882, the sensitivity was 80.6%, and the specificity was 83.0%.

**Table 1:** Test results regarding the detection of pneumothorax location in chest X-rays using fully-connected small artificial neural networks with a sigmoid activation function for all nodes.

Resolution	Hidden nodes	AUC	Cut-off	Sensitivity%	Specificity%	PPV%	NPV%	Accuracy%
20 × 20	49	0.876	0.122	78.3	84.2	37.5	97.0	83.6
30 × 30	49	0.881	0.101	81.0	83.6	37.4	97.3	83.3
20 × 20	49–49-49	0.876	0.084	82.0	80.7	34.0	97.4	80.8
30 × 30	49–49-49	0.882	0.101	80.6	83.0	36.5	97.2	82.7

Table 2 reports the pneumothorax detection results for each of the boxes in chest X-ray images of the test set with an input resolution of 256 × 256 pixels achieved by the CNN models including 1 or 2 hidden layers in the fully-connected part. For each of the fully-connected hidden nodes, a sigmoid or RELU activation function was applied. Comparing Tables 1 and 2, the diagnostic performance of the fully-connected small ANN was better than that of the CNN. As presented in Table 2, the performance of the CNN with the sigmoid activation function for fully-connected hidden nodes was compared better than that of the CNN with the RELU activation function [refer to Eq. (1)].

**Table 2:** Test results regarding the detection of pneumothorax location in chest X-rays using convolution neural networks with a sigmoid or RELU activation function for the fully-connected hidden nodes.

Activation function	Hidden nodes	AUC	Cut-off	Sensitivity%	Specificity%	PPV%	NPV%	Accuracy%
Sigmoid	49	0.861	0.119	76.6	81.1	33.0	96.6	80.6
Sigmoid	49–49	0.859	0.128	76.9	82.6	34.8	96.7	81.9
RELU	49	0.829	0.072	80.8	76.9	29.7	97.1	77.3
RELU	49–49	0.795	0.134	73.9	82.6	34.0	96.3	81.7

**Figure 3:**
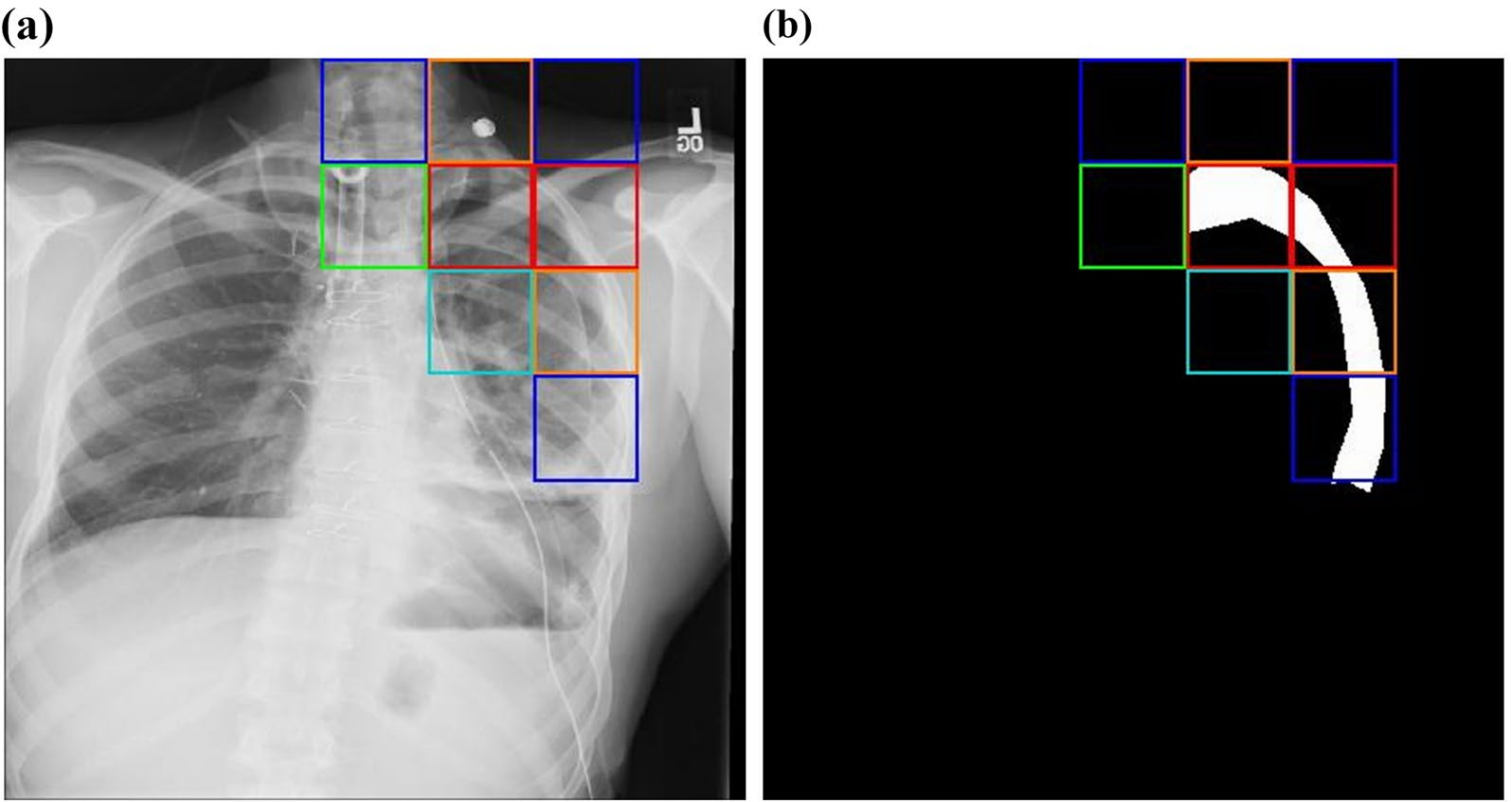
Example of well-predicted pneumothorax location. The location of pneumothorax predicted by a small artificial neural network is indicated by colored boxes. (**a**) Original chest X-ray image. (**b**) Image obtained after marking the location of pneumothorax in white and the remaining areas in black.

**Figure 4:**
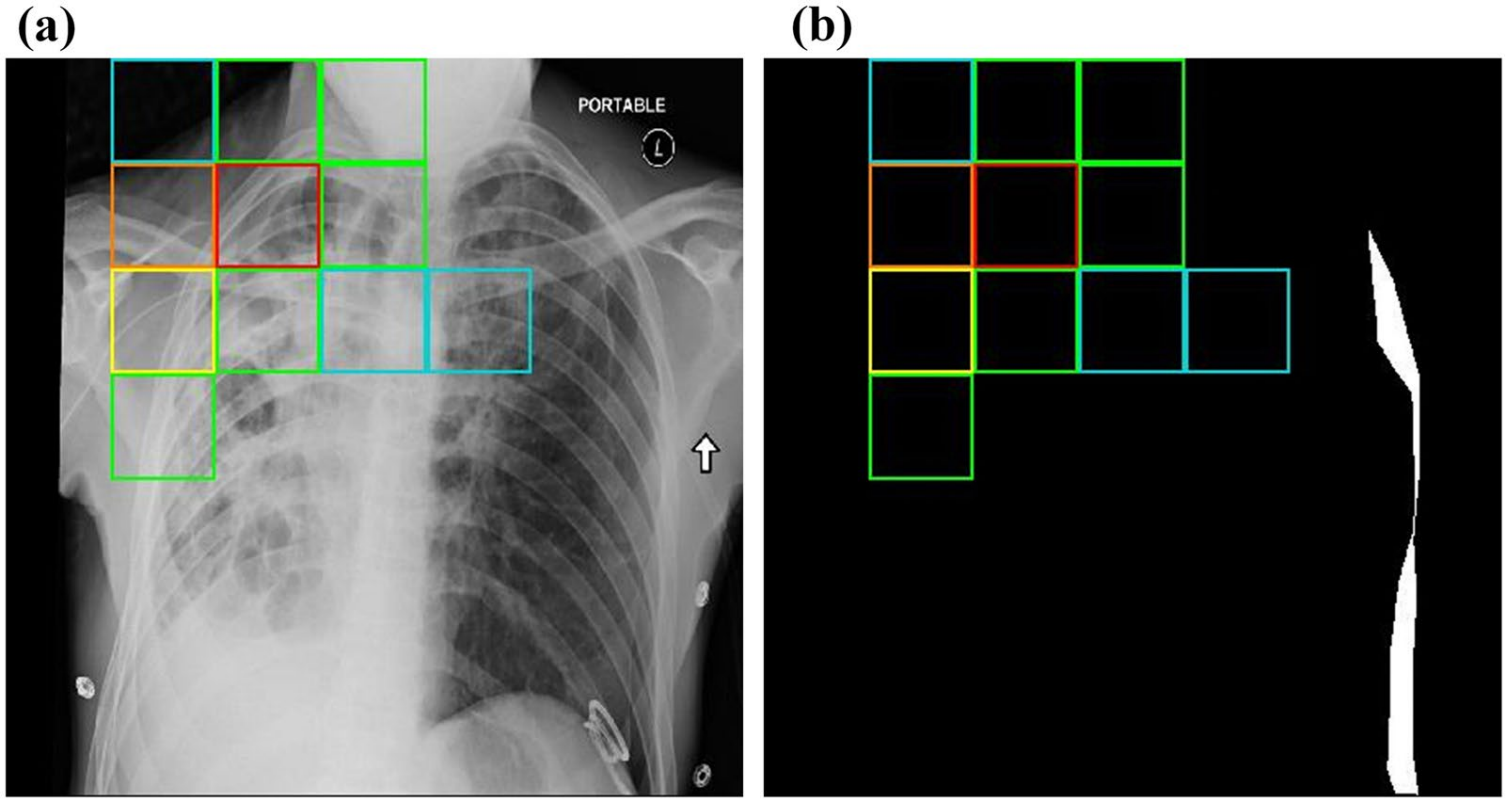
Example of inaccurately predicted pneumothorax location. The location of pneumothorax predicted by a small artificial neural network is indicated by colored boxes. (**a**) Original chest X-ray image. (**b**) Image obtained after marking the location of pneumothorax in white and the remaining areas in black.

Examples of the pneumothorax locations predicted by the best ANN model (i.e., the model with the input pixel resolution of 30 × 30 and 49–49–49 hidden nodes in Table 1) are indicated by colored boxes, as shown in Figs. 3 and 4. Each colored box indicates the location of the corresponding output node when the output value exceeded the cut-off value. The range from the cut-off value to 1.0 was divided into 6 sections, and the output value of each box was colored with 6 different colors from large to small: red, brown, yellow, green, blue, and cyan. Figure 3 shows an example of pneumothorax location that was properly predicted by the small ANN, where the boxes indicate that the predicted location completely overlap with the actual pneumothorax. Figure 4 shows an example of an inaccurately predicted case, where the actual pneumothorax location and the predicted boxes do not overlap at all.

## Discussion

We applied various fully-connected small ANN models for detecting the location of pneumothorax in chest X-ray images and obtained the best diagnostic performance when using an input pixel resolution of 30 × 30. The resolution of the input X-ray image used by the ANN affected its diagnostic performance in terms of pneumothorax localization. As the pixel resolution of the ANN input images increased from 20 × 20 pixels, its diagnostic performance for detecting the location of pneumothorax reached its highest value at 30 × 30 pixels and then degraded at higher resolutions. Therefore, rather than simply increasing the resolution of input images for ANNs, reducing the input resolution to an appropriate level could yield better results^16,18^.

Recently, several studies have been published regarding the prediction of pneumothorax location on chest X-ray images using deep learning^9–11^. One previous study performed pneumothorax segmentation using a framework combining UNet CNNs with various backbone networks^9^. The framework's performance, as measured by the Dice factor in the study, was 0.8574. In another study, a CNN called CheXLocNet had an intersection over union of 0.81 and a Dice score of 0.82 for automatic pneumothorax localization^10^. In another recent paper, the pneumothorax found in chest X-rays was segmented using a CNN called a fully convolutional DenseNet, achieving a mean pixelwise accuracy (MPA) of 0.93 and a Dice similarity coefficient of 0.92^11^. The outcome indicators of our study and these studies were different, and it was difficult to directly compare their performances with ours. However, we confirmed that the fully-connected small ANN automatically predicted the location of pneumothorax better than the CNN, which is a common deep-learning method currently used for image recognition.

A novel deep-learning algorithm^16^, a simple training process called the Kim-Monte Carlo algorithm, was applied to fully-connected small ANN models to detect the location of pneumothorax in chest X-rays, resulting in an AUC of 0.882, a sensitivity of 80.6%, and a specificity of 83.0% for an input resolution of 30 × 30 pixels, as shown in Table 1. When CNN was applied to the same dataset, the AUC was 0.861, the sensitivity was 76.6%, and the specificity was 81.1%, as shown in Table 2. Compared with the CNN, our approach used an extremely small ANN structure and a simple training process, and the resulting diagnostic performance was better than that of the CNN. Therefore, CNNs may not perform well for localization other than on classification or object detection tasks.

In addition, the diagnostic performance of the CNN with a sigmoid activation function for fully-connected hidden nodes was better than that of the CNN with RELU activation function, as presented in Table 2. As seen from Eq. (1), the RELU activation function might remove some important data flow information from the ANN. For the back-propagation method^13–15^, the RELU has been widely used as the activation function for fully-connected hidden nodes in ANNs due to its convenience when calculating the output gradient of a given node. However, a sigmoid activation function, which properly represents the characteristics of ANNs, should be used rather than the RELU activation function.

This approach can be used as an alternative to screening patients with chest X-rays, and it can provide quick responses to patients that are suspected of having pneumothorax while also being able to overcome the generally poor performance of human visual diagnostics, especially when the image quality is degraded due to various technical issues^18^. This study was the first attempt to detect pneumothorax in each of the divided boxes in chest X-ray images using deep-learning methods. The diagnostic performance of this study was very high. This deep-learning screening system will help clinicians classify patients who need further evaluation and urgent intervention. Our approach can help a primary physician screen for pneumothorax and significantly improve their ability to diagnose pneumothorax on chest X-rays in real emergency situations without expert assistance.

There are some limitations to this study. First, this study focused only on pneumothorax among various chest diseases. It will be interesting to examine the diagnostic performances of the deep-learning models for other diseases in the future. Second, the training and testing processes for the deep-learning models were conducted on a single public dataset. If external validation is performed in the future, the validity of these models for clinical applications will be further recognized. Third, it is necessary to validate how well our approach will perform in the real clinical field in the future. It will be one way to compare our approach with the reading of chest X-ray images by experts in various fields, including radiology and emergency medicine. Fourth, there are no computed tomography (CT) images in the NIH image dataset. It seems appropriate to conduct a new study using chest CT as the standard. Future studies using chest X-ray images confirmed by chest CT could validate our approach.

In conclusion, this study demonstrated that the performance of a small ANN in terms of detecting the location of pneumothorax was better than those of CNNs, and the CNN with a sigmoid activation function was better than the CNN with the RELU activation function. Finally, this study showed that our approach can accurately detect the location of pneumothorax in chest X-rays, significantly reduce the time delay incurred when diagnosing urgent diseases such as pneumothorax, and increase the effectiveness of clinical practice and patient care.

## Data Availability

The NIH Chest X-rays dataset used in this study can be downloaded from https://nihcc.app.box.com/v/ChestXray-NIHCC.

## References

[1] Bintcliffe O, Maskell N (2014). Spontaneous pneumothorax. BMJ.

[2] Wong A, Galiabovitch E, Bhagwat K (2019). Management of primary spontaneous pneumothorax: A review. ANZ J. Surg..

[3] O’Connor AR, Morgan WE (2005). Radiological review of pneumothorax. BMJ.

[4] Taylor AG, Mielke C, Mongan J (2018). Automated detection of moderate and large pneumothorax on frontal chest X-rays using deep convolutional neural networks: A retrospective study. PLoS Med..

[5] Hwang EJ (2020). Deep learning algorithm for surveillance of pneumothorax after lung biopsy: A multicenter diagnostic cohort study. Eur. Radiol..

[6] Park S (2019). Application of deep learning-based computer-aided detection system: Detecting pneumothorax on chest radiograph after biopsy. Eur. Radiol..

[7] Majkowska A (2020). Chest radiograph interpretation with deep learning models: Assessment with radiologist-adjudicated reference standards and population-adjusted evaluation. Radiology.

[8] Wang Y, Sun L, Jin Q (2019). Enhanced diagnosis of pneumothorax with an improved real-time augmentation for imbalanced chest X-rays data based on DCNN. IEEE/ACM Trans. Comput. Biol. Bioinform..

[9] Tolkachev A, Sirazitdinov I, Kholiavchenko M, Mustafaev T, Ibragimov B (2021). Deep learning for diagnosis and segmentation of pneumothorax: The results on the Kaggle competition and validation against radiologists. IEEE J. Biomed. Health Inform..

[10] Wang H, Gu H, Qin P, Wang J (2020). CheXLocNet: Automatic localization of pneumothorax in chest radiographs using deep convolutional neural networks. PLoS ONE.

[11] Wang Q (2020). Automated segmentation and diagnosis of pneumothorax on chest X-rays with fully convolutional multi-scale ScSE-DenseNet: A retrospective study. BMC Med. Inform. Decis. Mak..

[12] LeCun Y, Bengio Y, Hinton G (2015). Deep learning. Nature.

[13] Werbos, P. Beyond Regression: New Tools for Prediction and Analysis in the Behavioral Sciences. *Ph. D. dissertation, Harvard University* (1974).

[14] Sathyanarayana S (2014). A gentle introduction to backpropagation. Numeric Insight.

[15] Rumelhart DE, Hinton GE, Williams RJ (1986). Learning representations by back-propagating errors. Nature.

[16] Kim JS, Cho Y, Lim TH (2019). Prediction of the location of the glottis in laryngeal images by using a novel deep-learning algorithm. IEEE Access.

[17] Lee JY, Kim JS, Kim TY, Kim YS (2020). Detection and classification of intracranial haemorrhage on CT images using a novel deep-learning algorithm. Sci. Rep..

[18] Kim M, Kim JS, Lee C, Kang B-K (2021). Detection of pneumoperitoneum in the abdominal radiograph images using artificial neural networks. Eur. J. Radiol. Open.

[19] Hernández G, Zamora E, Sossa H (2020). Hybrid neural networks for big data classification. Neurocomputing.

[20] Gómez-Flores W, Sossa H (2021). Smooth dendrite morphological neurons. Neural Netw..

[21] Wang, X. *et al.* ChestX-Ray8: Hospital-Scale Chest X-Ray Database and Benchmarks on Weakly-Supervised Classification and Localization of Common Thorax Diseases. in *2017 IEEE Conference on Computer Vision and Pattern Recognition (CVPR)* 3462–3471. 10.1109/cvpr.2017.369 (2017).

[22] Filice RW (2020). Crowdsourcing pneumothorax annotations using machine learning annotations on the NIH chest X-ray dataset. J. Digit. Imaging.

